# Anlotinib-associated pulmonary embolism in brainstem glioblastoma treatment: a case report

**DOI:** 10.3389/fonc.2025.1526337

**Published:** 2025-04-09

**Authors:** Jia-Lan Zhao, Yong-Li Zhang, Ke-Jun Qu, Yang-Yang Jiang, Jiang-Lin Li, Jia Zhou, Shu-Teng Wu, Jun-Wei Li

**Affiliations:** ^1^ Department of Pharmacy, Shenzhen People’s Hospital (The Second Clinical Medical College, Jinan University, The First Affiliated Hospital, Southern University of Science and Technology), Shenzhen, China; ^2^ Department of Pathology, Shenzhen People’s Hospital (The Second Clinical Medical College, Jinan University, The First Affiliated Hospital, Southern University of Science and Technology), Shenzhen, China

**Keywords:** anlotinib, glioblastoma, pulmonary embolism, targeted therapy, biopsy

## Abstract

**Background:**

Glioblastoma (GBM) is the most common and aggressive primary brain malignancy in adults. Diagnosis primarily relies on imaging techniques like CT scan and MRI, while pathological biopsy remains the diagnostic gold standard. Standard of care for newly diagnosed GBM includes maximal safe resection followed by radiotherapy and chemotherapy, although prognosis remains poor. GBM patients are at heightened risk for venous thromboembolism (VTE), including deep vein thrombosis (DVT) and pulmonary embolism (PE), with chemotherapy and targeted therapy further elevating this risk.

**Case summary:**

We report a case of a patient with atypical cranial imaging findings, where initial assessments at both an external hospital and our institution were equivocal. A definitive GBM diagnosis was achieved only after biopsy. GBMs are highly vascularized malignant tumors. Anlotinib, an anti-angiogenic multi-kinase inhibitor, has been used to treat GBM. Following diagnosis, the patient received anlotinib therapy and subsequently developed PE, suspected as an anlotinib-induced adverse event.

**Conclusion:**

Anlotinib may cause PE and should be used with caution. Clinicians should close coagulation monitoring following anlotinib treatment, including D-dimer testing and imaging (eg, CT), to ensure prompt diagnosis and timely treatment for PE. This case highlights the critical need for vigilant PE monitoring and prompt management in GBM patients on anlotinib therapy.

## 1 Introduction

Glioblastoma (GBM), a high-grade glioma, is the most aggressive primary brain tumor in adults, classified as grade IV by the WHO (1–3). Diagnosis relies heavily on imaging—primarily CT and MRI—followed by histopathological and molecular analyses from tumor biopsy or resection to confirm grade and subtype (3). This diagnostic process can be protracted, delaying treatment. Standard of care for GBM includes maximal safe surgical resection, followed by radiotherapy and chemotherapy, though prognosis remains poor, with a five-year survival rate around 6% (3, 4). GBM patients also face an elevated risk of venous thromboembolism (VTE), including deep vein thrombosis (DVT) and pulmonary embolism (PE), risks exacerbated by chemotherapy and targeted therapy (5, 6). We report on a case of a patient with atypical neuroimaging findings and a protracted diagnostic course, ultimately diagnosed with GBM after biopsy and treated with anlotinib, after which the patient developed PE. The case details are as follows.

## 2 Case report

**Figure 1:**
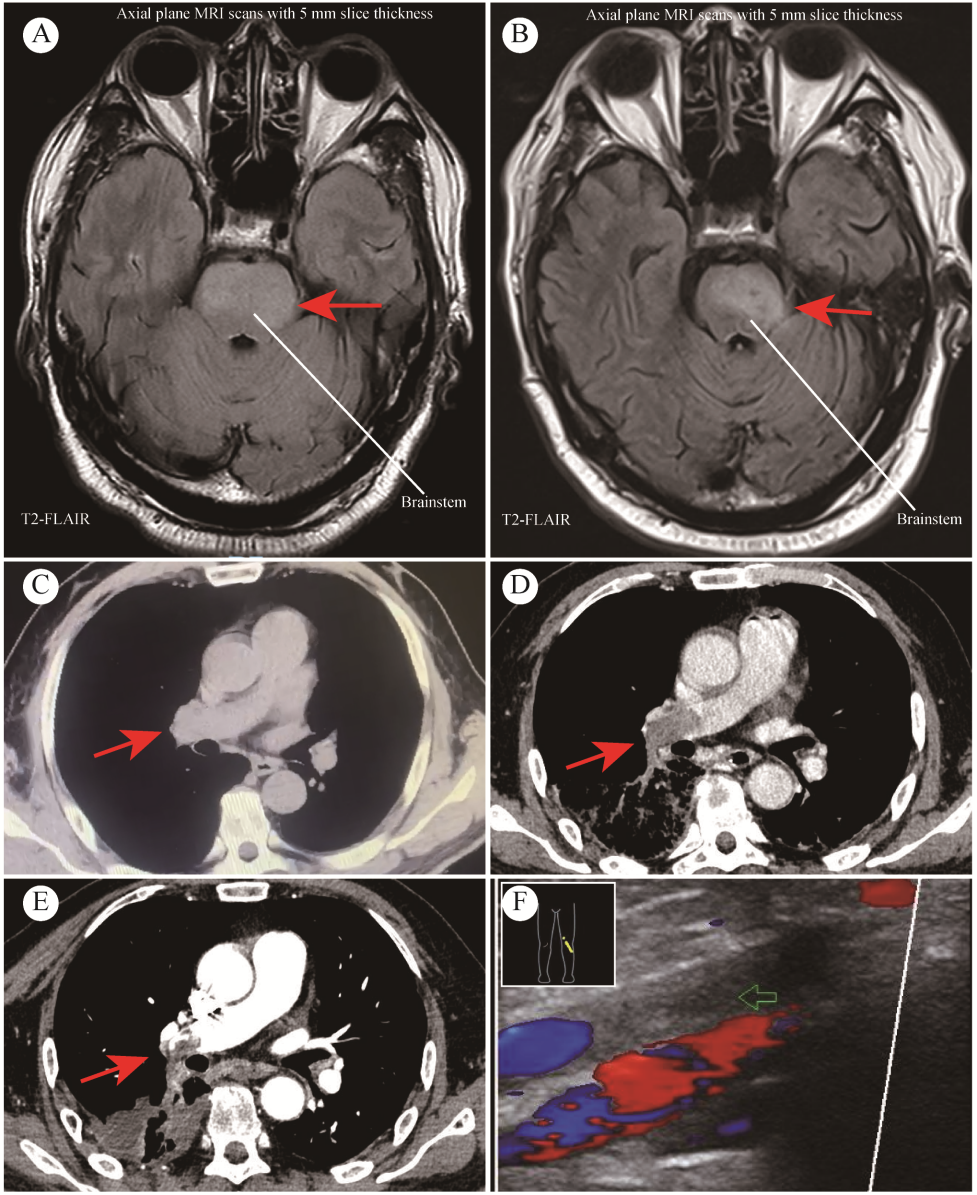
MRI and CT Imaging from case study. **(A)** Preoperative MRI findings; **(B)** MRI findings after 3 months of anlotinib treatment; **(C)** CT indicated no PE before initiating anlotinib treatment; **(D)** CT indicated extensive PE after 3 months of anlotinib treatment; **(E)** CT scan after 2 months of anticoagulant therapy; **(F)** Thromboses detected in lower leg veins.

A 64-year-old Asian male presented with recurrent right-sided weakness in September 2023. His history was unremarkable for major diseases. Initial cranial CT showed mild cerebral atrophy, and MRI indicated infiltrative lesions in the brain (**Figure 1A**), suggestive of inflammation or low-grade glioma. Treated with butylphthalide, edaravone, aspirin, and clopidogrel, his symptoms persisted. At our neurosurgery department, extensive testing, including lumbar puncture and whole-body PET/CT, failed to reveal a definitive diagnosis. Stereotactic brainstem biopsy performed in October 2023 confirmed GBM (WHO Grade IV, IDH wild-type). The molecular pathology showed *IDH1* wild-type, *TERT* promoter mutation, and *CDKN2A* homozygous deletion. The immunohistochemistry showed Oligo-2 (+), P53 (–), ATRX (+, no loss), GFAP (+), KI-67 (approximately 80%+), Vim (focal+), H3-K27M (-), IDH-1 (-), MGMT (-), Syn (partially+). The patient had an Eastern Cooperative Oncology Group (ECOG) performance status score of 1.The patient received radiotherapy (DT60Gy) with concurrent anlotinib (12 mg daily for two weeks on, one week off). After 3 months of anlotinib treatment, MRI indicated disease progression (**Figure 1B**), and anlotinib was discontinued in February 2024.

**Figure 2:**
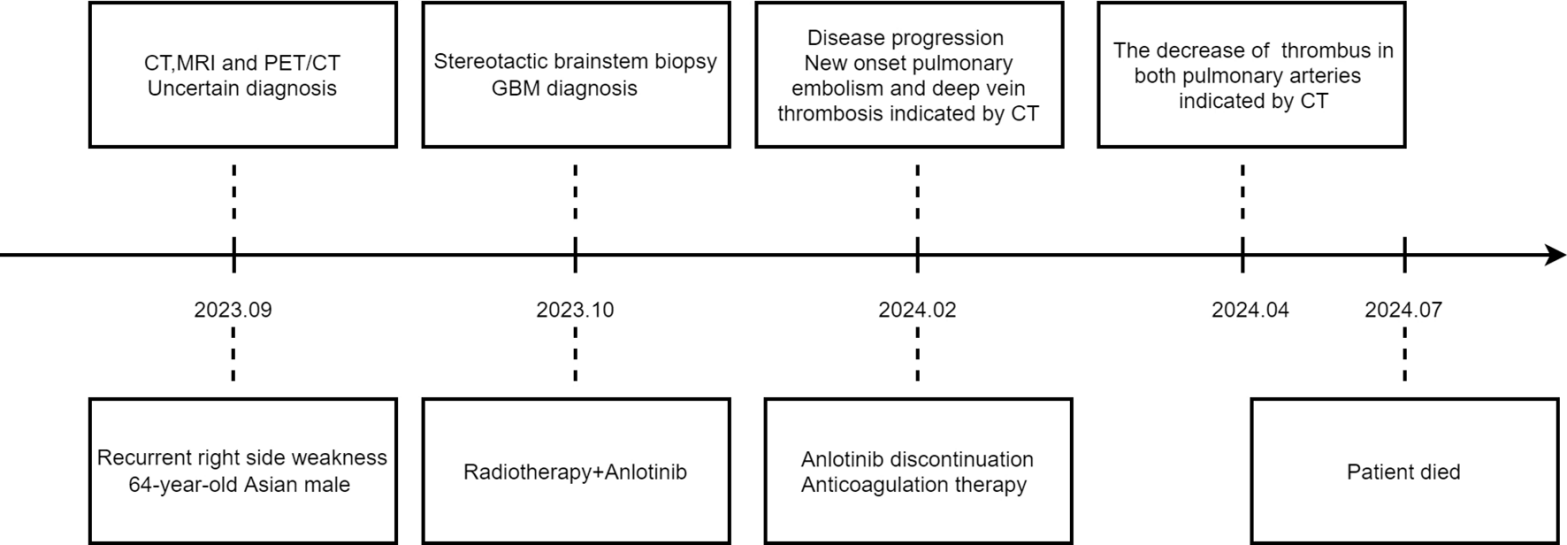
Timeline of each treatment.

At the same time, he was confirmed by CT of thromboses in both pulmonary arteries (**Figure 1D**), and with associated deep vein thrombosis in the lower extremities (**Figure 1F**). Before initiating anlotinib treatment, CT revealed no PE occurred (**Figure 1C**). Key laboratory test results were demonstrated as follows: oxygen saturation (SpO_2_) 97.7% when oxygen therapy, platelet count (PLT) 208×10^9/L, D-dimer 3.61↑μg/L, antithrombin (AT) 56%↓. Anticoagulation therapy was initiated, shifting from heparin (due to heparin resistance) to argatroban. But the treatment effect was suboptimal and he was given fondaparinux. After 2 months of anticoagulant treatment, the patient CT scan showed the thrombus in the left pulmonary artery had almost completely disappeared, and the thrombus in the right pulmonary artery had decreased (**Figure 1E**). Anlotinib initially achieved disease stabilization; however, the subsequent PE significantly compromised the patient’s functional status and overall well-being. Despite aggressive treatment, he experienced further complications and passed away five months post-diagnosis. The timeline is shown in **Figure 2**.

## 3 Discussion

This case illustrates the diagnostic challenges in atypical GBM presentations and highlights the limitations of imaging alone. When GBM exhibits atypical imaging characteristics, including morphology, lesion number, location, signal intensity, it is prone to misdiagnosis as other diseases. In this report, the tumor’s brainstem location and overlapping MRI signal characteristics delayed the diagnosis, ultimately confirmed through biopsy, the diagnostic gold standard, though it imposes substantial physical, psychological, and economic burdens on patients.

The current standard of care for GBM is the Stupp regimen—surgical resection followed by radiotherapy with concurrent and adjuvant temozolomide (TMZ)—yet median progression-free survival (PFS) is 6.9 months, and median overall survival (OS) is 14.6 months (3, 4, 7). Novel approaches, including targeted therapy(eg, angiogenesis inhibitors), immunotherapy(eg, immune checkpoint blockade), and physical therapy (eg, Tumor Treating Fields, TTF) hold promise but are largely experimental (8, 9). GBMs are highly vascularized malignant tumors that produce VEGF (7, 10). Robust aberrant angiogenesis renders GBM potentially amenable to anti-angiogenic therapy (7, 10). Anlotinib, an anti-angiogenic multi-kinase inhibitor, has been used to treat GBM. Our patient’s tumor was IDH wild-type, MGMT promoter unmethylated and TERT-mutated suggesting a poor prognosis, particularly given MGMT promoter unmethylation, indicating limited TMZ efficacy. For such patients, clinical trials or alternative regimens may be prioritized. The patient received radiotherapy with concurrent and adjuvant anlotinib.

Anlotinib, a tyrosine kinase inhibitor (TKI), targets onco-angiogenesis and suppresses tumor growth by simultaneously blocking vascular endothelial growth factor receptor (VEGFR)1/2/3, platelet-derived growth factor receptor (PDGFR), fibroblast growth factor receptor (FGFR), and c-kit (11). Li et al. discovered that anlotinib effectively inactivated the JAK3/STAT3 pathway to inhibit growth and induce apoptosis in malignant glioma cells independent of MGMT expression. Meanwhile, anlotinib alone or in combination with radiation was effective and safe *in vivo* evaluation (12). A phase II study of anlotinib combined with the Stupp regimen in patients with newly diagnosed GBM reported the median PFS was 10.9 months, median OS was17.4 months, and the 12 months PFS rate was 48.5% (7). Anlotinib has shown efficacy in GBM, though its role in increasing VTE risk warrants attention. While VEGFR TKIs are generally safe, cases of PE have been reported in cancer patients receiving anlotinib combination treatment. These cases underscore a possible link between VEGF inhibition, endothelial dysfunction, and thrombogenesis (6, 13–16). The mechanism underlying anlotinib-induced VTE may involve the inhibition of VEGF signaling pathway, leading to endothelial dysfunction, decreased production of nitric oxide and prostacyclin, increased endothelin-1 production, which facilitate platelet aggregation, thereby increasing the risk of VTE (6, 15). Additionally, angiogenesis inhibitors induce an increase in tissue factor levels and are associated with an increased incidence of VTE (6).

Managing VTE in GBM patients is challenging. The high intrinsic risk of VTE complicates treatment, especially with tumor-associated venous thromboembolism (TAVTE). Studies have shown that the risk of VTE in cancer patients is 9-fold higher than in general population (17). The occurrence of VTE has been reported to increase the likelihood of death for cancer patients by 2- to 6-fold (17, 18). Notably, the incidence rate of VTE is 39% in GBM patients, significantly higher than other tumor types and is correlated with an elevated risk of intracranial hemorrhage (5).Besides cancer-related factors, both chemotherapy and targeted therapy are associated with an increased risk of VTE in cancer patients (6). Guidelines regarding the treatment of VTE in GBM patients are lacking. The current management for VTE in patients with GBM follows the established guidelines for VTE in patients with other tumor types. There is a CSCO guideline for VTE prophylaxis (19). The guideline does not recommend routine anticoagulation for VTE prophylaxis in all cancer patients. However, pharmacological thromboprophylaxis may be offered in high-risk case such as medical treatment patients with cancer who have acute medical illness or reduced mobility, and high-risk cancer patients (Khorana score of 2 or higher). Moreover, Current guidelines recommend anticoagulation for VTE treatment, yet heparin resistance in low antithrombin (AT) conditions, as seen in this patient, necessitates alternatives like argatroban or direct thrombin inhibitors (17, 18, 20, 21).

It is necessary to strengthen awareness and management of VTE following angiogenesis inhibitors treatment for GBM, especially in patients with comorbidities such as cardiovascular diseases or a prior history of VTE (5, 15, 22). Neuro-oncologists close coagulation monitoring and early anticoagulation could prevent treatment discontinuation due to VTE adverse event. Moreover, a close collaboration between neuro-oncologists and cardiovascular specialists should be emphasized.

## 4 Conclusion

This case underscores the critical role of biopsy in GBM diagnosis, particularly in cases with atypical imaging findings. Despite a comprehensive treatment approach, GBM remains highly aggressive, with limited treatment efficacy and survival gains. Early, accurate diagnosis and multimodal treatment are essential for improving outcomes. Anlotinib may cause PE and should be used with caution. Therefore, Clinicians should have a high index of suspicion for prompt diagnosis and timely treatment. Meanwhile, use of anlotinib in patients with a prior history of thrombotic events or other known risk factors for VTE warrants careful consideration. The therapeutic potential of anlotinib in GBM requires validation through large-scale clinical trials, particularly to elucidate its role in VTE risk.

## Data Availability

The original contributions presented in the study are included in the article/supplementary material. Further inquiries can be directed to the corresponding author.
